# Survival in endometrial cancer in relation to minimally invasive surgery or open surgery – a Swedish Gynecologic Cancer Group (SweGCG) study

**DOI:** 10.1186/s12885-021-08289-3

**Published:** 2021-06-02

**Authors:** Christer Borgfeldt, Erik Holmberg, Janusz Marcickiewicz, Karin Stålberg, Bengt Tholander, Elisabeth Åvall Lundqvist, Angelique Flöter-Rådestad, Maria Bjurberg, Pernilla Dahm-Kähler, Kristina Hellman, Elisabet Hjerpe, Preben Kjölhede, Per Rosenberg, Thomas Högberg

**Affiliations:** 1 Department of Obstetrics and Gynecology, Skåne University Hospital, Lund University, SE-22185 Lund, Sweden; 2 Region Västra Götaland, Regional Cancer Centre West, SE-41345 Gothenburg, Sweden; 3 Department of Obstetrics and Gynecology, Halland Hospital, SE-43281 Varberg, Sweden; 4 Department of Women’s and Children’s Health, Uppsala University, SE-75185 Uppsala, Sweden; 5 Department of Oncology, Uppsala University Hospital, SE-75185 Uppsala, Sweden; 6 Department of Oncology and Department of Biomedical and Clinical Sciences, Linköping University, SE-58185 Linköping, Sweden; 7 Department of Women’s and Children’s Health, Division of Neonatology, Obstetrics and Gynecology, Karolinska Institutet, Karolinska University Hospital, SE-17176 Stockholm, Sweden; 8 Department of Hematology, Oncology and Radiation Physics, Skåne University Hospital, and Department of Clinical Sciences, Lund University, SE-22185 Lund, Sweden; 9 Department of Obstetrics and Gynecology, Institute of Clinical Sciences, Sahlgrenska Academy, SE-41345 Gothenburg, Sweden; 10 Department of Gynecologic Cancer, Theme Cancer, Karolinska University Hospital, SE-171 76 Stockholm, Sweden; 11 Department of Gynecology and Obstetrics, Visby Hospital, SE-62155 Visby, Sweden; 12 Department of Obstetrics and Gynecology in Linköping, Department of Biomedical and Clinical Sciences, Linköping University, SE-58185 Linköping, Sweden; 13 Department of Medical Oncology, Department of Clinical Sciences, Lund University, SE-22100 Lund, Sweden

**Keywords:** Endometrial cancer, Minimally invasive surgery, Survival, Risk factors

## Abstract

**Background:**

The aim of this study was to analyze overall survival in endometrial cancer patients’ FIGO stages I-III in relation to surgical approach; minimally invasive (MIS) or open surgery (laparotomy).

**Methods:**

A population-based retrospective study of 7275 endometrial cancer patients included in the Swedish Quality Registry for Gynecologic Cancer diagnosed from 2010 to 2018. Cox proportional hazard models were used in univariable and multivariable survival analyses.

**Results:**

In univariable analysis open surgery was associated with worse overall survival compared with MIS hazard ratio, HR, 1.39 (95% CI 1.18–1.63) while in the multivariable analysis, surgical approach (MIS vs open surgery) was not associated with overall survival after adjustment for known risk factors (HR 1.12, 95% CI 0.95–1.32). Higher FIGO stage, non-endometrioid histology, non-diploid tumors, lymphovascular space invasion and increasing age were independent risk factors for overall survival.

**Conclusion:**

The minimal invasive or open surgical approach did not show any impact on survival for patients with endometrial cancer stages I-III when known prognostic risk factors were included in the multivariable analyses.

**Supplementary Information:**

The online version contains supplementary material available at 10.1186/s12885-021-08289-3.

## Synopsis highlights

The minimal invasive or open surgical approach did not show any impact on survival for patients with endometrial cancer stages I-III when adjusting for FIGO stage, morphology, ploidy, lymphovascular space involvement and age.

Independent risk factors for overall survival were higher FIGO stage, non-endometrioid histology, non-diploid tumors, lymphovascular space invasion and increasing age.

## Background

Endometrial cancer is the most common gynecologic cancer in developed countries. The median age at onset is around 70 years and very few women are affected before the age of 50. There is strong evidence that the rising incidence seen throughout the Western world is associated with lifestyle factors, such as obesity, diabetes mellitus, late menopause, and an aging population [1, 2]. The treatment for presumed early-stage endometrial cancer involves surgery removing the uterus and performing a bilateral salpingo-oophorectomy with or without lymphadenectomy followed by radiotherapy and/or chemotherapy in selected cases based upon the estimated risk for recurrence or death. Minimally invasive surgery (MIS) in patients with endometrial cancer reduces morbidity, the time needed to resume normal activities of daily living, the number of days before return to work, length of hospital stay, and blood loss in patients with and without lymph node dissection, especially in elderly and overweight patients [3–6]. Randomized trials have shown that total laparoscopic hysterectomy seems to be equally safe as total abdominal hysterectomy, but population-based studies are needed to confirm these results [7, 8]. After the randomized controlled “LACC trial” and registry studies in cervical cancer were published, the MIS approach in cervical cancer has been disapproved in several countries, and in most national guidelines open surgery is recommended [9, 10]. Concerning long-term survival outcomes after MIS in endometrial cancer patients’ further studies are needed. The aim of this study was to analyze overall survival in patients with endometrial cancer surgical FIGO stage I-III in the whole Swedish population in relation to surgical approach, MIS vs open surgery (laparotomy) adjusting in multivariable analyses for known prognostic factors.

## Methods

Reporting to the Swedish National Cancer Registry (NCR), which started in 1958, is mandatory for both pathologists and clinicians, and the registry has over 95% coverage for all malignant tumors, of which 99% are histologically verified. The Swedish Quality Registry for Gynecologic Cancer (SQRGC) started the registration of endometrial cancer in 2010. The registration is web-based and includes information on patient and tumor characteristics, treatment details and follow-up.

Reporting to the SQRGC is performed prospectively by all hospitals and clinics in the six Swedish health care regions. Quality control is continuously performed by registrars at the regional cancer centers who monitor entered data. Through the personal identification numbers allocated to all citizens in Sweden, the SQRGC continuously receives date of death from the Population Registry enabling coverage control compared to the NCR and life-long follow-up of patients. The validity of SQRGC data has been assessed, with 70–100% agreement between registered data and the original case files [11]. Every patient can choose to opt out of registration.

### Study population

The SQRGC was used to identify patients with endometrial cancer (ICD-10 code C54) stage I-III diagnosed from January 1st, 2010 through December 31st, 2018. Inclusion criteria were age at least 18 years, histologically verified primary endometrial cancer treated with MIS or open surgical approach, endometrioid-, serous-, mucinous-, clear cell carcinomas and carcinosarcoma morphology (*n* = 12,582). Exclusion criteria were endometrial cancer FIGO stage IV (*n* = 295) and sarcomas (*n* = 586). According to the Swedish National Guidelines endometrial cancer stage IV surgery shall be performed by laparotomy. The coverage between the SQRGC and the NCR was checked and showed agreement in 97–100% (personal communication). Surgical staging was performed according to the Federation Internationale de Gynecologie et d’Obstetrique (FIGO) classification from 2009 [12]. There were exclusions due to missing data on FIGO stage (*n* = 1378), missing data on risk factors (*n* = 1475), no primary surgery (*n* = 228) or surgical approach (*n* = 1931). The final data set included 7275 patients.

Most of the patients in the study were treated according to the Swedish National Guidelines for Endometrial cancer from 2011 (the guidelines were updated in 2017). In the guidelines, preoperative high-risk was defined as non-endometrioid histology (serous, clear cell carcinoma or carcinosarcoma), endometrioid adenocarcinoma FIGO grade 3, or non-diploid tumors. In preoperative high-risk tumors, a lymphadenectomy of the pelvic and para-aortic regions (up to the left renal vein) was recommended in addition to hysterectomy and salpingo-oophorectomy. Preoperative evaluation of myometrial infiltration was not included as a criterion for lymphadenectomy in the national guidelines until the revised version of 2017, when also analysis of DNA-ploidy was abandoned. Postoperatively high-risk patients in FIGO stage I-II were defined as those with non-endometrioid histology or those with endometrioid histology with two or more risk factors; grade 3, ≥50% myometrial invasion or non-diploid tumor. Patients allotted to the postoperative high-risk group in FIGO stage I-II were recommended chemotherapy ± brachytherapy and those with positive lymph nodes (FIGO stage III) or no lymphadenectomy were offered chemotherapy ± external radiotherapy. Women with preoperative signs of advanced disease (FIGO stage III) were surgically treated with the intension to obtain macroscopic radicality. Lymphovascular space involvement (LVSI) was not included as a high-risk parameter in either the first or in the revised versions.

Most DNA analyses were performed by flow cytometry and a minority by image cytometry [13, 14]. Positive LVSI was defined as obvious lymphovascular space invasion identified in routine hematoxylin and eosin staining in accordance with the Swedish Society of Pathology guidelines for endometrial cancer. There is no requirement for foci to be confirmed with immunohistochemistry, only that the pathologist making the diagnosis judges the focus to be sufficiently clear to be diagnostic.

Patients were followed until 15 March 2019 or to emigration or death, whichever came first.

The ethical review board at Gothenburg University approved the study (Dnr 814–15).

### Statistics

Distributions of descriptive data in Table 1 were compared between groups using Pearson’s chi-square test and Fisher’s exact tests for categorical variables, and Student’s *t* test and Wilcoxon rank-sum test for continuous variables, as appropriate. The main outcome was overall survival (OS) measured from the date of diagnosis to the date of the first event of death, emigration, or end of follow-up (March 15th, 2019). OS probabilities were calculated using the Kaplan–Meier method. The Cox proportional hazard models were used in uni- and multivariable survival analyses. The multivariable analyses included type of surgery, morphology, FIGO stage, grade of the endometrioid carcinomas, ploidy, LVSI and age at diagnosis. Hazard ratios (HR) with 95% confidence intervals (CI) were reported for 5 years follow-up period. The proportional hazard assumption was checked using Schoenfeld’s residuals. When the assumption was violated (indicated in the tables) the HR was interpreted as the mean over the 5-year follow-up period. All comparisons were two-sided, and a 5% level of significance was used. All statistical analyses were carried out with Stata/IC 16.1 for Mac (StataCorp. 2020. Stata: Release 16. Statistical Software. College Station, TX: StataCorp LLC).

**Table 1:** Patients’ characteristics

Variable	MIS ***N*** = 3742	Open surgery ***N*** = 3533	Total ***N*** = 7275	p
Age, mean (min-max)	68.6 (25–98)	68.5 (29–97)	68.5 (25–98)	0.93^a^
Age, median (25–75%)	69 (62–76)	69 (62–76)	69 (62–76)	0.78^b^
Age, years				0.95^c^
0–59	718 (19.2%)	696 (19.7%)	1414 (19.4%)	
60–69	1185 (31.7%)	1110 (31.4%)	2295 (31.6%)	
70–79	1261 (33.7%)	1190 (33.7%)	2451 (33.7%)	
≥ 80	578 (15.4%)	572 (15.2%)	1115 (15.3%)	
WHO performance status				0.002^d^
0	2230 (59.6%)	1453 (41.1%)	3683 (50.6%)	
1	789 (21.1%)	634 (18.0%)	1423 (19.6%)	
2	175 (4.7%)	127 (3.6%)	302 (4.2%)	
3	20 (0.5%)	23 (0.6%)	43 (0.6%)	
4	2 (0.0%)	5 (0.1%)	7 (0.1%)	
Missing	526 (14.1%)	1291 (36.5%)	1817 (25.0%)	
FIGO stage				< 0.001^c^
IA	2481 (66.3%)	1925 (54.5%)	4406 (60.6%)	
IB	744 (19.9%)	783 (22.2%)	1527 (21.0%)	
II	249 (6.6%)	315 (8.9%)	564 (7.8%)	
III	268 (7.2%)	510 (14.4%)	778 (10.7%)	
Morphology				< 0.001^c^
Endometrioid	3426 (91.6%)	2960 (83.8%)	6386 (87.8%)	
FIGO grade 1–2	2976 (86.9%)	2281 (77.1%)	5257 (82.3%)	
FIGO grade 3	362 (10.6%)	573 (19.4%)	935 (14.6%)	
Missing	88 (2.6%)	106 (3.6%)	194 (3.0%)	
Serous	196 (5.2%)	299 (8.5%)	495 (6.8%)	
Clear cell	67 (1.8%)	108 (3.1%)	175 (2.4%)	
Carcinosarcoma	53 (1.4%)	166 (4.7%)	219 (3.0%)	
Postoperative risk groups				< 0.001^c^
Low	2299 (61.4%)	1642 (46.5%)	3941 (54.2%)	
High	1443 (38.6%)	1891 (53.5%)	3334 (45.8%)	
LVSI				< 0.001^c^
No	2547 (68.1%)	2051 (58.0%)	4598 (63.2%)	
Yes	464 (12.4%)	646 (18.3%)	1110 (15.3%)	
Missing	731 (19.5%)	836 (23.7%)	1567 (21.5%)	
Ploidy				< 0.001^c^
Diploid	2414 (64.5%)	2005 (56.8%)	4419 (60.7%)	
Non-diploid	719 (19.2%)	947 (26.8%)	1666 (22.9%)	
Missing	609 (16.3%)	581 (16.4%)	1190 (16.4%)	
Follow-up (years), median (25–75%)				
Censored	3.7 (2.0–5.4)	5.1 (3.2–7.1)	4.3 (2.4–6.1)	
Death	2.3 (1.4–3.8)	2.6 (1.3–4.3)	2.5 (1.3–4.1)	
All	3.5 (1.9–5.2)	4.6 (2.6–6.6)	4.0 (2.2–5.9)	

*FIGO* International Federtion of Gynecology and Obstetics (Federation Internationale de Gynecologie et d’Obstetrique)

*LVSI* Lymphovascular space involvement

^a^Student’s t-test

^b^Wilcoxon rank-sum test

^c^Pearson’s chi-squared test

^d^Fisher’s exact test

## Results

The median age in both the MIS and open surgery (laparotomy) groups was 69 (range 25–98) years (Table 1). The percentage of patients with functional status 2–4 (WHO-status) was similar in both groups. The median follow-up time in the MIS group was 3.5 years and in the open surgery group 4.6 years.

### Survival analyses

**Fig. 1:**
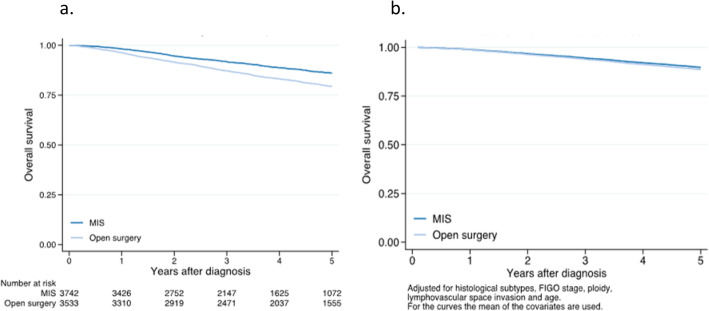
**a** Overall survival estimates for minimally invasive surgery (MIS) (reference) and open surgery (laparotomy), hazard ratio 1.57 (95% confidence interval 1.38–1.79) in the univariable Cox proportional hazards model. **b** Overall survival estimates for minimally invasive surgery (MIS) (reference) and open surgery (laparotomy) adjusted for morphology, FIGO stage, ploidy, lymphovascular space invasion, and age. Hazard ratio was non significant in the multivariable Cox proportional hazards model

In the univariable Cox analysis, open surgery was associated with worse survival compared with MIS (HR 1.39 95% CI 1.18–1.63) (Fig. 1a, Table 2). In the multivariable analysis including all endometrial cancer with MIS vs. open surgery, endometrioid vs. non-endometrioid tumors, FIGO stage (IA vs. IB vs. II vs. III), diploid vs. non-diploid tumors, LVSI yes vs. no, and age groups, there was no statistically significant difference between open surgery and MIS (HR 1.12 95% CI 0.95–1.32) (Fig. 1b, Table 2,). Since grade is only related to endometrioid endometrial cancer, it was not included in this multivariable analysis. Non-endometrioid tumor, non-diploid tumor, LVSI, myometrial invasion (FIGO stage IA vs IB), FIGO stage and increasing age were all independent risk factors (Table 2).

**Table 2:** Endometrial cancer, FIGO stage I-III. Uni- and multivariable Cox proportional hazard regression analyzing overall survival including type of surgery, morphology, FIGO stage, ploidy, lymphovascular space invasion and age

Variables	No. of patients	Univariable Cox regression		Multivariable Cox regression	
		HR (95% CI)	p	HR (95% CI)	p
Surgical approach					
MIS	2527	Ref.		Ref.	
Open surgery	2286	1.39 (1.18–1.63)	< 0.001	1.12 (0.95–1.32)	0.19
Endometrioid					
Yes	4378	Ref.		Ref.	
No	435	3.91 (3.24–4.71)	< 0.001	1.75 (1.42–2.18)	< 0.001
FIGO stage^a^					
IA	2978	Ref.		Ref.	
IB	1019	2.09 (1.69–2.58)	< 0.001	1.32 (1.06–1.65)	0.013
II	359	3.07 (2.36–3.99)	< 0.001	1.81 (1.38–2.38)	< 0.001
III	457	6.25 (5.10–7.67)	< 0.001	2.74 (2.16–3.49)	< 0.001
Ploidy					
Diploid	3534	Ref.		Ref	
Non-diploid	1279	2.75 (2.34–3.23)	< 0.001	1.59 (1.33–1.91)	< 0.001
LVSI					
No	3896	Ref.		Ref.	
Yes	917	4.24 (3.61–4.98)	< 0.001	2.25 (1.86–2.72)	< 0.001
Age group (years)					
0–59	943	Ref.		Ref.	
60–69	1535	2.11 (1.44–3.10)	< 0.001	1.88 (1.28–2.76)	0.001
70–79	1624	4.40 (3.08–6.31)	< 0.001	3.66 (2.55–5.26)	< 0.001
80-	711	10.7 (7.44–15.3)	< 0.001	8.65 (6.00–12.5)	< 0.001

^a^The proportional hazard rates assumption is not fulfilled

In the corresponding uni- and multivariable analysis of endometrioid endometrial cancer, where FIGO grade 1 + 2 vs 3 was added and endometrioid vs. non-endometrioid tumors was omitted, there was no statistically significant difference between MIS and open surgery (Table 3). All the other included risk factors carried independent prognostic information (Table 3). In addition, surgical approach was not an independent variable among non-endometrioid carcinomas (Table 4). FIGO stage, morphology, and age groups were all independent prognostic factors (Table 4).

**Table 3:** Endometrioid endometrial carcinoma, FIGO stage I-III. Uni- and multivariable Cox proportional hazard regression analyzing overall survival including type of surgery, FIGO grade, FIGO stage, ploidy, lymphovascular space invasion and age

Variables	No. of patients	Univariable Cox regression		Multivariable Cox regression	
		HR (95% CI)	p	HR (95% CI)	p
Surgical approach					
MIS	2310	Ref.		Ref.	
Open surgery	1950	1.33 (1.10–1.61)	0.003	1.12 (0.93–1.36)	0.24
FIGO grade^a^					
1–2	3621	Ref.		Ref.	
3	639	2.47 (2.01–3.05)	< 0.001	1.31 (1.02–1.66)	0.031
FIGO stage^a^					
IA	2720	Ref.		Ref.	
IB	942	2.22 (1.77–2.79)	< 0.001	1.34 (1.05–1.71)	0.019
II	295	3.36 (1.70–3.28)	< 0.001	1.55 (1.10–2.18)	0.012
III	303	5.18 (3.99–6.72)	< 0.001	2.60 (1.92–3.53)	< 0.001
Ploidy					
Diploid	3395	Ref.		Ref.	
Non-diploid	865	2.01 (1.64–2.45)	< 0.001	1.43 (1.15–1.78)	0.001
LVSI^a^					
No	3544	Ref.		Ref.	
Yes	716	3.64 (3.00–4.42)	< 0.001	2.08 (1.66–2.61)	< 0.001
Age group (years)					
0–59	886	Ref.		Ref.	
60–69	1374	1.94 (1.26–3.00)	0.003	1.94 (1.26–3.00)	0.003
70–79	1405	4.10 (2.73–6.17)	< 0.001	3.82 (2.54–5.76)	< 0.001
80-	595	10.8 (7.18–16.2)	< 0.001	9.6 (6.39–14.5)	< 0.001

^a^The proportional hazard rates assumption is not fulfilled

**Table 4:** Non-endometrioid endometrial carcinoma, FIGO stage I-III. Uni- and multivariable Cox proportional hazard regression analyzing overall survival including type of surgery, subtype, FIGO stage, and age

Variables	No. of patients	Univariable Cox regression		Multivariable Cox regression	
		HR (95% CI)	p	HR (95% CI)	p
Surgical approach					
MIS	316	Ref.		Ref.	
Open surgery	573	1.10 (0.86–1.42)	0.44	0.98 (0.76–1.27)	0.88
Morphology^a^					
Clear cell	175	0.73 (0.52–1.03)	0.076	0.68 (0.48–0.96)	0.030
Serous	495	Ref.		Ref.	
Carcinosarcoma	219	1.77 (1.38–2.27)	< 0.001	1.67 (1.29–2.16)	< 0.001
FIGO stage					
IA	372	Ref.		Ref.	
IB	143	2.26 (1.54–3.32)	< 0.001	1.99 (1.34–2.93)	0.001
II	117	3.70 (2.57–5.34)	< 0.001	3.47 (2.40–5.03)	< 0.001
III	257	4.50 (3.30–6.12)	< 0.001	4.68 (3.42–6.40)	< 0.001
Age group (years)					
0–59	74	Ref.		Ref.	
60–69	256	1.43 (0.79–2.60)	0.24	1.67 (0.92–3.04)	0.093
70–79	359	2.18 (1.23–3.87)	0.008	2.50 (1.41–4.46)	0.002
80-	200	4.17 (2.34–7.43)	< 0.001	4.74 (2.65–8.50)	< 0.001

^a^The proportional hazard rates assumption is not fulfilled

In node positive patients surgical approach showed no association with OS (Table 5) Finally, the analysis comparing open surgery and robotic-assisted laparoscopy and conventional laparoscopy separately, showed no association between surgical approach and OS (Table 6).

**Table 5:** Endometrial cancer, in patients with metastases in lymph nodes. Uni- and multivariable Cox proportional hazard regression analyzing overall survival including type of surgery, morphology, ploidy, lymphovascular space invasion and age

Variables	No. of patients	Univariable Cox regression		Multivariable Cox regression	
		HR (95% CI)	p	HR (95% CI)	p
Surgical approach					
MIS	64	Ref.		Ref.	
Open surgery	137	1.31 (0.70–2.45)	0.40	1.36 (0.72–2.57)	0.34
Endometrioid					
Yes	132	Ref.		Ref.	
No	69	2.81 (1.63–4.85)	< 0.001	1.87 (1.04–3.38)	0.037
Ploidy					
Diploid	118	Ref.		Ref	
Non-diploid	83	2.65 (1.36–5.14)	0.004	2.19 (1.09–4.40)	0.029
LVSI					
No	61	Ref.		Ref.	
Yes	140	3.11 (1.41–6.90)	0.005	3.10 (1.38–6.97)	0.006
Age group (years)					
0–59	43	Ref.		Ref.	
60–69	72	1.28 (0.53–3.11)	0.59	1.33 (0.55–3.24)	0.53
70–79	68	2.84 (1.22–6.60)	0.015	2.60 (1.11–6.12)	0.028
80-	18	3.08 (1.03–9.19)	0.043	3.73 (1.22–11.4)	0.021

**Table 6:** Endometrial cancer. Uni- and multivariable Cox proportional hazard regression analyzing overall survival including type of surgery, morphology, FIGO stage, ploidy, lymphovascular space invasion and age

Variables	No. of patients	Univariable Cox regression		Multivariable Cox regression	
		HR (95% CI)	p	HR (95% CI)	p
Surgical approach					
Robotic laparoscopy	1986	Ref.		Ref.	
Laparoscopy	541	0.78 (0.57–1.07)	0.12	1.03 (0.74–1.42)	0.87
Open surgery	2286	1.31 (1.11–1.56)	0.002	1.12 (0.94–1.34)	0.20
Endometrioid					
Yes	4378	Ref.		Ref.	
No	435	3.91 (3.24–4.71)	< 0.001	1.75 (1.42–2.18)	< 0.001
FIGO stage					
IA	2978	Ref.		Ref.	
IB	1019	2.09 (1.69–2.58)	< 0.001	1.32 (1.06–1.66)	0.013
II	359	3.06 (2.35–3.97)	< 0.001	1.81 (1.38–2.39)	< 0.001
III	457	6.25 (5.10–7.67)	< 0.001	2.75 (2.16–3.49)	< 0.001
Ploidy					
Diploid	3534	Ref.		Ref	
Non-diploid	1279	2.75 (2.34–3.23)	< 0.001	1.59 (1.33–1.91)	< 0.001
LVSI^a^					
No	3896	Ref.		Ref.	
Yes	917	4.24 (3.61–4.98)	< 0.001	2.25 (1.86–2.72)	< 0.001
Age group (years)					
0–59	943	Ref.		Ref.	
60–69	1535	2.11 (1.44–3.10)	< 0.001	1.88 (1.28–2.76)	0.001
70–79	1624	4.40 (3.07–6.31)	< 0.001	3.66 (2.55–5.26)	< 0.001
80-	711	10.7 (7.44–15.3)	< 0.001	8.65 (6.00–12.4)	< 0.001

^a^The proportional hazard rates assumption is not fulfilled

## Discussion

In this large population-based registry study in endometrial cancer patients, minimally invasive and open surgery showed no overall survival difference when known prognostic factors were included in the multivariable analyses. Independent risk factors for worse overall survival were FIGO stage, non-endometrioid histology, non-diploid tumors, lymphovascular space invasion, increasing age, and, in endometrioid tumors, FIGO grade 3.

A Danish nationwide registry study showed that the overall survival among women with early-stage endometrial cancer was improved after the introduction of robot-assisted MIS, and that MIS was associated with a lower mortality rate compared with laparotomy even after adjustment for histopathological risk groups [15]. These results are in contrast with our findings. We found no impact of surgical approach on overall survival. In comparison with the Danish study, we included more prognostic factors in the regression models i.e. DNA- ploidy and lymphovascular space involvement. This demonstrates the importance to include known risk factors reducing bias in observational studies. Even if our study did not show any difference in overall survival, MIS has shown to be associated with reduced surgical morbidity and faster recovery, especially in overweight patients [3–6]. Although laparoscopic robot-assisted surgery has been demonstrated to have a shorter learning curve than laparoscopic surgery, several studies still indicate that approximately 50 laparoscopic robot-assisted surgery hysterectomies must be performed to gain proficiency [16, 17]. MIS may be used as a proxy parameter in quality measurement in endometrial cancer surgery and MIS should be accomplished in more than 80% of the patients in high volume centers [18].

A US register study including 6304 elderly (65+) middle-class women with early-stage endometrial cancer enrolled in a US national insurance program (Medicare) found a tendency towards better overall survival in favor of MIS compared with laparotomy [19]. A recent meta-analysis including six published randomized controlled trials with 3993 patients demonstrated that overall survival after MIS was similar to the overall survival after open surgery and that MIS was associated with reduced surgical morbidity, which also has been shown in a Swedish study in elderly patients [6, 20]. Socioeconomic conditions and surgical allocation may affect survival. However, in the Danish study socioeconomic status did not affect the outcome. The Danish health care system is comparable to the Swedish system. Since no difference in overall survival between MIS and open surgery could be demonstrated and there are less complications in MIS, MIS should be advocated in elderly to facilitate and improve recovery.

Most endometrial cancers are diagnosed in an early stage and the RCTs included only presumed early FIGO stage patients. However, the finding of lymph node metastases render a higher final stage. In the RCT study by Walker at al., 14% of patients were in FIGO stages III-IV, and in this population-based cohort 11% were in stage III (*n* = 778). In some of the other RCTs the number of patients was very small in the advanced stages, so no comparisons were possible between MIS and open surgery. In our population cohort there was a high number of patients in stage III and we did not find any difference in survival outcome between MIS and open surgery in stage III (data not shown).

FIGO grade 3 endometrioid endometrial cancer is preoperatively classified as high-risk endometrial cancer since there is an increased risk for lymph node metastasis, therefore international and Swedish national guidelines recommend systematic pelvic and para-aortic lymph node dissection. In our material, FIGO grade 3 endometrioid carcinomas also showed significantly worse overall survival in the multivariable analysis.

The non-endometrioid carcinomas are known to have a worse prognosis than the endometrioid carcinomas, therefore in most guidelines they are classified as high-risk and adjuvant therapy with chemo- +/− radiotherapy is recommended [21]. The non-endometrioid carcinomas are often included in randomized controlled endometrial cancer studies even though it is often not possible to analyze the subtypes separately due to their low number [7]. This large study included 924 patients with non-endometrioid carcinomas, MIS and laparotomy showed no difference in the multivariable analysis. Carcinosarcoma showed the worst prognosis similar to a smaller Canadian study [22]. Clear cell carcinomas showed the best prognosis of the non-endometrioid tumors in line with an American study [23].

Our group previously reported LVSI as an independent risk factor for lymph node metastases and decreased survival in patients with endometrioid adenocarcinomas [24]. In this material we also included the non-endometrioid carcinomas where, in the multivariable analyses, we found LVSI to be an independent prognostic factor for decreased overall survival in endometrial cancer stage I-III. Available evidence suggests that LVSI in the primary tumor may serve as a marker for both lymphatic and hematologic dissemination [25, 26].

This nationwide population-based registry study using prospectively collected data may include selection bias. Women with a large uterus that may contain a larger tumor as well as obvious enlarged lymph nodes on CT scan may have been selected for open surgery. The health care system is relatively uniform all over Sweden and is free of charge to all citizens living in Sweden. The studies in the Cochrane meta-analysis included five RCTs with 3993 patients in the primary outcome overall survival and only one study finished after 2013 [20]. Our single registry study had no exclusion criteria, almost twice as many patients as the Cochrane meta-analysis and was run in a population-based manner including all patients in the regular national health care system. In Sweden almost no cancer patients receive treatment by private health care providers. Large register studies such as the present and the Danish study by Jorgensen et al. [15] including confounders in the multivariable analyses add knowledge how the surgical or any treatment modality work in populations in the regular health care system.

There are some limitations to be considered e.g. that the Swedish Guidelines for Endometrial cancer published in 2011 included recommendations for lymph node staging and the procedure was gradually implemented, which is why not all grade 3 endometrioid endometrial cancer patients or patients with high-risk histology had a lymphadenectomy performed during the beginning of the study period, perhaps leading to under-staging. Moreover, not all patients had LVSI analyzed. The registry includes no molecular analyses which should be considered in the future. However, the number of patients with LVSI analyzed was high: 4813 in the multivariable analyses. Robot-assisted surgery has been introduced the last 10 years and it is performed mostly at tertiary centers by experienced surgeons. This may have improved the outcome in the MIS group. During the last 2 years of the study period the sentinel lymph node concept was introduced, and some university centers also used this technique in low-risk endometrial cancer patients. For all patients with high-risk endometrial cancer, however, the recommendation of full lymph node staging up to the renal veins remained. It is unknown how sentinel lymph node mapping will impact patient outcomes, including the need for adjuvant postoperative radio- and/or chemotherapy, as well as the incidence of adverse events and survival. Future analyses concerning oncological outcomes after the implementation of sentinel lymph node procedures are needed to evaluate the long-term effects.

## Conclusion

In this large population-based study including over 7000 patients with endometrial cancer stages I-III, surgical approach, MIS or open surgery, had no influence on overall survival when adjustment was made for known prognostic factors.

## Supplementary Information

**Additional file 1: Supplementary Table 1.** Endometrial cancer. Uni- and multivariable Cox proportional hazard regression analyzing overall survival including type of surgery, morphology, lymph node metastases, ploidy, lymphovascular space invasion and age.

**Additional file 2: Supplementary Table 2.** Separate uni- and multivariable Cox proportional hazard regression analyses for the specific histologies/morphologies performed for each morphology.

## Data Availability

All data and the material are available at the Regional Cancer Center West where the Swedish Quality Registry for Gynecologic Cancer (SQRGC) is administrated and located.

## Abbreviations

FIGO: The Federation Internationale de Gynecologie et d’Obstetrique
MIS: Minimally invasive surgery
NCR: Swedish National Cancer Registry
SQRGC: The Swedish Quality Registry for Gynecologic Cancer
LVSI: Lymphovascular space involvement
CI: Confidence intervals
WHO: World Health Organization
OS: Overall survival
DNA: Deoxyribonucleic acid
US: United States of America
RCT: Randomized controlled trial
