# Supplementary material for: Survival in endometrial cancer in relation to minimally invasive surgery or open surgery – a Swedish Gynecologic Cancer Group (SweGCG) study
Source: BMC Cancer. 2021 Jun 2;21:658. doi: 10.1186/s12885-021-08289-3 (PMC8170953; doi:10.1186/s12885-021-08289-3)
Supplement: Supplementary file 1 — Additional file 1: Supplementary Table 1. Endometrial cancer. Uni- and multivariable Cox proportional hazard regression analyzing overall survival including type of surgery, morphology, lymph node metastases, ploidy, lymphovascular space invasion and age. [file 12885_2021_8289_MOESM1_ESM.docx]

Supplementary table 1. Endometrial cancer. Uni- and multivariable Cox proportional hazard regression analyzing overall survival including type of surgery, morphology, lymph node metastases, ploidy, lymphovascular space invasion and age.

| Variables | No. of  patients | Univariable  Cox regression | | Multivariable  Cox regression | |
| --- | --- | --- | --- | --- | --- |
|  |  | HR (95% CI) | p | HR (95% CI) | p |
| Surgical approach  MIS  Open surgery | 627  734 | Ref.  1.15 (0.82-1.62) | 0.42 | Ref.  1.06 (0.75-1.50) | 0.74 |
| Endometrioid  Yes  No | 1103  258 | Ref.  3.24 (2.35-4.48) | <0.001 | Ref.  1.92 (1.35-2.72) | <0.001 |
| Lymph nodal  Negative  Positive | 1160  201 | Ref.  3.43 (2.45-4.80) | <0.001 | Ref.  1.88 (1.29-2.73) | 0.001 |
| Ploidy  Diploid  Non-diploid | 656  705 | Ref.  2.79 (1.93-4.04) | <0.001 | Ref  2.02 (1.37-2.96) | <0.001 |
| LVSI  No  Yes | 982  379 | Ref.  3.98 (2.88-5.52) | <0.001 | Ref.  2.84 (1.99-4.06) | <0.001 |
| Age group (years)  0-59  60-69  70-79  80- | 274  475  514  98 | Ref.  2.36 (1.28-4.35)  3.64 (2.01-6.59)  5.36 (2.60-11.0) | 0.006  <0.001  <0.001 | Ref.  2.14 (1.16-3.95)  3.23 (1.78-5.88)  4.52 (2.18-9.37) | 0.014  <0.001  <0.001 |
