# Supplementary material for: Survival in endometrial cancer in relation to minimally invasive surgery or open surgery – a Swedish Gynecologic Cancer Group (SweGCG) study
Source: BMC Cancer. 2021 Jun 2;21:658. doi: 10.1186/s12885-021-08289-3 (PMC8170953; doi:10.1186/s12885-021-08289-3)
Supplement: Supplementary file 2 — Additional file 2: Supplementary Table 2. Separate uni- and multivariable Cox proportional hazard regression analyses for the specific histologies/morphologies performed for each morphology. [file 12885_2021_8289_MOESM2_ESM.docx]

Supplementary table 2. Separate uni- and multivariable Cox proportional hazard regression analyses for the specific histologies/morphologies performed for each morphology

| Surgical approach  by morphologie | No. of  patients | Univariable  Cox regression | | Multivariable  Cox regression | |
| --- | --- | --- | --- | --- | --- |
|  |  | HR (95% CI) | p | HR (95% CI) | p |
| Surgical approach^1^  MIS  Open surgery | 2310  1950 | Ref.  1.33 (1.10-1.61) | 0.003 | Ref.  1.12 (0.93-1.36) | 0.24 |
| Surgical approach^2^  MIS  Open surgery | 153  196 | Ref.  1.51 (0.91-2.49) | 0.11 | Ref.  1.56 (0.92-2.65) | 0.10 |
| Surgical approach^3^  MIS  Open surgery | 51  79 | Ref.  0.84 (0.38-1.88) | 0.67 | Ref.  1.00 (0.44-2.26) | 0.99 |
| Surgical approach^4^  MIS  Open surgery | 38  115 | Ref.  0.56 (0.33-0.94) | 0.028 | Ref.  0.70 (0.41-1.20) | 0.20 |

^1.^ Endometrioid. Adjusted mutivariable for histological grade, FIGO stage, Ploidy, LVSI and age.

^2.^ Serous carcinoma. Adjusted mutivariable for FIGO stage and age.

^3.^ Clear cell carcinoma. Adjusted mutivariable for FIGO stage and age.

^4.^ Carcinosarcoma. Adjusted mutivariable for FIGO stage and age.
